# Hepatic Arterial Infusion of Chemotherapy for Advanced Hepatobiliary Cancers: State of the Art

**DOI:** 10.3390/cancers13123091

**Published:** 2021-06-21

**Authors:** Carmelo Laface, Mariarita Laforgia, Pasquale Molinari, Ippazio Ugenti, Cosmo Damiano Gadaleta, Camillo Porta, Girolamo Ranieri

**Affiliations:** 1 Interventional and Medical Oncology Unit, IRCCS Istituto Tumori “Giovanni Paolo II”, Viale Orazio Flacco 65, 70124 Bari, Italy; c.laface@studenti.uniba.it (C.L.); p.molinari@oncologico.bari.it (P.M.); c.gadaleta@oncologico.bari.it (C.D.G.); 2 Department of Biomedical Sciences and Clinical Oncology, University of Bari Aldo Moro, 70124 Bari, Italy; camillo.porta@uniba.it; 3 Pharmacy Unit, IRCCS Istituto Tumori “Giovanni Paolo II”, Viale Orazio Flacco 65, 70124 Bari, Italy; m.laforgia@oncologico.bari.it; 4 Department of Emergency and Organ Transplantation, University Aldo Moro of Bari, 70124 Bari, Italy; ippazio.ugenti@uniba.it; 5 Division of Digestive Endoscopy, IRCCS Istituto Tumori “Giovanni Paolo II”, Viale Orazio Flacco 65, 70124 Bari, Italy

**Keywords:** hepatocarcinoma, cholangiocarcinoma, hepatic arterial infusion chemotherapy, implanted pump or port

## Abstract

**Simple Summary:**

Liver functional failure is one of the leading causes of cancer-related death. Systemic chemotherapy usually offers a modest benefit in terms of disease control rate, progression-free survival, and overall survival at the cost of a significant percentage of adverse events. Liver malignancies are mostly perfused by the hepatic artery while the normal liver parenchyma by the portal vein network. On these bases, the therapeutic strategy consisting of hepatic arterial infusion of chemotherapy takes place. This review aims to summarize the current knowledge on this approach from different points of view, such as techniques, drugs pharmacology and pharmacokinetics, and clinical outcomes for advanced hepatobiliary cancers. Most of the collected studies have several limitations: non-randomized retrospective design, a relatively small number of patients, the hepatic arterial administration of different chemotherapeutic agents, as well as its combination with a great heterogeneity of systemic agents. However, despite these limitations, the presented data show favorable results in terms of safety and efficacy for hepatic arterial infusion of chemotherapy, with respect or in alternative to the gold standard treatment, even when they are combined with systemic treatments. Therefore, this therapeutic strategy may be an alternative or an integrative treatment option for advanced hepatobiliary cancers. Further and larger prospective, randomized, multi-center studies, with well-defined inclusion criteria and treatment strategies, are required to confirm the presented data.

**Abstract:**

Liver functional failure is one of the leading causes of cancer-related death. Primary liver tumors grow up mainly in the liver, and thus happens for liver metastases deriving from other organs having a lower burden of disease at the primary site. Systemic chemotherapy usually offers a modest benefit in terms of disease control rate, progression-free survival, and overall survival at the cost of a significant percentage of adverse events. Liver malignancies are mostly perfused by the hepatic artery while the normal liver parenchyma by the portal vein network. On these bases, the therapeutic strategy consisting of hepatic arterial infusion (HAI) of chemotherapy takes place. In literature, HAI chemotherapy was applied for the treatment of advanced hepatobiliary cancers with encouraging results. Different chemotherapeutic agents were used such as Oxaliplatin, Cisplatin, Gemcitabine, Floxuridine, 5-Fluorouracil, Epirubicin, individually or in combination. However, the efficacy of this treatment strategy remains controversial. Therefore, this review aims to summarize the current knowledge on this approach from different points of view, such as techniques, drugs pharmacology and pharmacokinetics, and clinical outcomes for advanced hepatobiliary cancers.

## 1. Introduction

Liver functional failure is one of the most frequent and critical causes of cancer-related morbidity and death. Primary liver tumors grow up mainly in the liver, and thus happens for liver metastases deriving from other organs having a lower burden of disease at the primary site [1,2,3].

Normal liver parenchyma is one of the most sprinkled body districts, having two distinct blood sources: The portal vein flow supplying approximately 75% of liver perfusion and the hepatic arterial flow. In the case of primary and secondary liver tumors, hepatic artery becomes the main source of blood favoring tumor growth [4]. This is the reason why hepatic arterial infusion (HAI) delivering chemotherapeutic agents selectively within liver parenchyma, through a catheter or pump, is able to attack directly local disease, while sparing healthy liver and minimizing systemic toxicity. The choice of the appropriate chemotherapeutic drug for HAI lies in its pharmacokinetic properties:

- Large first-pass extraction so that a significant percentage is extracted by the liver, achieving a high drug concentration within the intrahepatic circulation;
- Short plasma half-life to avoid systemic accumulation;
- High total body clearance.

A prolonged drug exposure by means of a continuous infusion system can increase cell killing, thanks to the drug persistence in the therapeutic window. These conditions allow increasing the local drug action on liver malignancies and limiting systemic toxicity, but they cannot substitute in toto the efficacy of systemic chemotherapy, which still remains the principal approach in several types of cancers. In light of this, the combination of HAI and systemic chemotherapy, as documented in several studies, represents a winning strategy for diffuse liver primary and secondary disease.

Hepatobiliary cancers can be distinguished in hepatocellular carcinoma (HCC) and bile ducts tumors. HCC is the most frequent primary liver cancer, the sixth common cancer and the fourth leading cause of cancer-related deaths in the world. Almost 25% of HCC patients present an advanced setting at the diagnosis and approximatively 50% develop hepatic metastases during disease evolution. Bile ducts tumors are the second most frequent primary liver cancer counting for 15% of all. They are a heterogeneous group of highly malignant tumors, including intrahepatic carcinoma of the gallbladder and ampulla of Vater (ICC), extrahepatic hilar (Klatskin–Altemeier tumor), and distal extrahepatic cholangiocarcinomas. The ICC incidence is 20% of all cholangiocarcinomas [5]. 

Advanced hepatobiliary cancers can show different clinical manifestations, such as multifocal tumor hepatic spread, major vessel invasion, and/or extrahepatic metastasis, but all share a poor prognosis in the advanced setting, with a median overall survival (OS) of 5–12 months. Moreover, the few available systemic therapies offer only a modest efficacy compared to their related toxicity. Sorafenib, Lenvatinib, Cabozantinib, and Regorafenib are the principal target drugs approved for the treatment of advanced HCC. They are all tyrosine kinase inhibitors (TKIs), blocking different kinase enzymes involved in several pro-tumoral cellular pathways inducing uncontrolled proliferation and angiogenesis. In particular, Sorafenib interacts with BRAF, BRAF^V600E^, cKIT, and FLT3 on tumor cells and VEGFR2–3 and PDGFRβ on tumor endothelial cells [6]. Lenvatinib blocks the cascade of VEGFR1–2–3, FGFR1–2–3–4, PDGFRα, cKIT, and RET activation [7]; Cabozantinib inhibits the cellular pathways of MET, VEGF, RET, ROS1, KIT, FLT3, and TIE-2; Regorafenib acts as inhibitor of VEGFR1–2–3, TIE-2, KIT, RET, BRAF^V600E^, PDGFR, and FGFR [8].

In addition, FDA has approved checkpoint immunotherapeutics, Nivolumab in 2017 and Pembrolizumab in 2018, in second-line treatment for advanced HCC resistant to Sorafenib, but until now neither EMA nor AIFA have released authorization for this therapeutic use. Immunotherapy disengages the block of immune T-cells induced by the tumor, by linking the surface PD-1 receptor and avoiding its interaction with the natural ligand PD-L1. Subsequently, immune response against the tumor can take place [9].

Among the drugs in clinical trials for a second-line treatment, Ramucirumab has also been demonstrated to be effective in particular clinical conditions [10]; Ramucirumab is a direct VEGFR2 antagonist, binding VEGFR2 and blocking its interaction with natural VEGFR ligands (VEGF-A, VEGF-C, and VEGF-D), secreted by solid tumors to promote angiogenesis and enhance tumor blood supply [11].

Sorafenib was tested in many clinical trials as a first-line treatment, demonstrating a weak prolonging OS time (2.3–2.8 months) and improving the response rate (RR, almost 3%). An OS of 3.1–6.0 months was evidenced for HCC patients with major portal vein tumor thrombosis (PVTT) [12]. Lenvatinib was compared to Sorafenib as a first-line treatment in a phase III clinical trial evidencing its non-inferiority in OS [13]. Cabozantinib was tested as a second-line treatment in the CELESTIAL trial, resulting in longer OS (10.2 months vs. 8.0 months for placebo; hazard ratio for death, 0.76; *p* = 0.005), progression-free survival (PFS, 5.2 months vs. 1.9 months; *p* < 0.001), and RR (4% vs. less than 1%; *p* = 0.009) [14]. The RESORCE phase III clinical trial compared Regorafenib to placebo in patients with progression disease during the Sorafenib treatment, showing an improvement of median OS (10.6 months vs. 7.8 months; *p* < 0.0001) [15]. 

The REACH phase III randomized trial evaluated Ramucirumab as a second-line therapy following Sorafenib, reporting an improvement for median PFS (*p* < 0.001) and time to progression (TTP, *p* < 0.001) but not for median OS, compared to the placebo [16]. The following REACH-2 randomized phase III trial documented that PFS and OS were longer in HCC patients who received Ramucirumab after Sorafenib and with a baseline AFP level of ≥400 ng/mL [10]. 

Nivolumab and Pembrolizumab, the first anti-PD-1 antibodies, were evaluated within phase I/II studies in advanced HCC patients who progressed on or were intolerant to Sorafenib, reporting modest clinical results on OS and ORR [17]. 

With regards to bile ducts tumors, Gemcitabine plus platin-based chemotherapy is still the unique therapy with evidence of clinical benefit, counting for a median OS of 11.7 months with respect to 8.1 months in the case of single agent gemcitabine [18]. 

The poor prognosis, the biological features of advanced hepatobiliary cancers, the double vascular network physiology of the healthy and cancerous liver, and the few available therapeutical strategies justify the big clinical need for integrative approaches, in particular for locoregional therapies. HAI chemotherapy, with or without combined systemic treatments represents a well-known route of administration but the choice of the best drug presupposes a deep knowledge of its molecular and pharmacokinetic properties [19,20,21,22,23,24]. This review aims at summarizing the current knowledge on the clinical approach of intrahepatic arterial drug administration in its different aspects of techniques, drug pharmacodynamics and pharmacokinetics, and clinical outcomes for advanced hepatobiliary cancers.

## 2. Hepatic Arterial Infusion Technical Procedure

Several HAI chemotherapy courses can be performed through the placement of a specific port-a-cath system. In the past, the infusion catheter was surgically implanted using a laparotomy but the patients were exposed to the risks associated both with laparotomy and general anesthesia. Moreover, the surgical technique had a high rate of complications often requiring further surgery to repair or replace the malfunctioning port-a-cath system [25]. In more recent decades, significant advances in interventional oncology have succeeded in the minimally invasive percutaneous implantation of specific port-a-cath systems by a simpler, safer, and faster technique without laparotomy or general anesthesia [26]. However, also the interventional approach may be difficult and risky, in particular in the clinical cases of variant hepatic arterial anatomy [27]. For this reason, the operators must be very experienced to reduce the risk of failure. Several minimally invasive percutaneous techniques of port-a-cath systems implantation are described to date and each of them is associated with different rates of procedure-related complications [28,29,30].

Catheter insertion is usually performed under local anaesthesia with lidocaine 1% 10–15 ml administered in the arterial puncture and port-a-cath subcutaneous pocket sites [31,32,33]. Long-acting analgesic agents such as morphine or fentanyl are used if local anaesthesia is inadequate to control pain [34,35]. Conscious sedation with benzodiazepines such as midazolam is usually reserved for anxious patients [31].

The Seldinger technique is the best-known percutaneous procedure, in the placement of infusion catheters to reach the hepatic artery. The left subclavian and femoral artery represent the most frequent peripheral accesses, though the hypogastric, subclavian, and brachial arteries have all been used, as well [36,37,38,39]. The increased risk of cerebral complications using brachial, axillary, and subclavian arterial accesses is inappropriate in clinics [40]. On the contrary, the common femoral arterial access is technically easier as the vessel is superficial and less tortuous [41].

The precise placement of the catheter tip is essential to perfuse the liver avoiding extrahepatic drug infusion. Since some chemotherapeutics scatter gastric or duodenal mucosal lesions and local irritation into the adjacent organs, by means of the arteries originating from the common hepatic artery (gastroduodenal artery and the right gastric artery in particular), hepatic arterial and celiac trunk anatomy are complex due to the great number of anatomical variants [42]. Therefore, an accurate and detailed hepatic arterial anatomy study, employing non-invasive computed tomography angiography (CTA) or magnetic resonance angiography (MRA) techniques, is necessary before inserting the infusion catheter [43]. Subsequently, results from this evaluation are confirmed with the two-dimensional (2 D) digital subtraction angiography (DSA) [43]. Nowadays, this technique has been supplemented with a three-dimensional (3 D) cone beam CT (CBCT) that includes opacification of vessels by making the detection of segmental and subsegmental arteries supplying the target tumor easier [44]. In addition, several studies have been evaluating with favourable results, the role of automatic feeder detection (AFD) software in the analyzation and digital process of CBCT imaging for simplifying and shortening the procedures of hepatic arterial port-a-cath system implantation [44,45,46,47,48]. Possible causes of failure of port-a-cath placement must be considered in this preliminary phase to evaluate if the patient is a suitable candidate for HAI. The most common conditions that can be a cause of port-a-cath placement failure are: Multiple hepatic arterial variants with a high risk of thrombosis, celiac artery stenosis due to median arcuate ligament compression, tumor invasion of target arteries, and fibrosis of both groins secondary to femoro-femoral bypass surgery [43].

The final position of the infusion catheter tip is usually based on the vascular anatomy of each patient and location of hepatic tumor lesions, in accordance with the preliminary angiographic study. Initially, percutaneously port-a-cath implantation consisted of placing the distal tip of the catheter into the common or proper hepatic artery. However, this approach was associated with a high rate of complications, such as catheter dislocation and hepatic artery thrombosis, potentially responsible for a temporary or permanent interruption of HAI chemotherapy [49,50,51]. Hepatic arterial thrombosis was generally due to the mechanical stimulation of the vascular endothelium by the catheter tip. This first approach has been modified over time with the "fix-catheter-tip" technique, where the distal tip of the catheter has been fixed to the gastroduodenal artery, while the injected drug flows into the proper hepatic artery through a side hole [52]. Another catheter placement technique is the "long tapered catheter placement", in which the catheter is positioned, but not fixed, as distally as possible into the common hepatic artery with the side hole placed at the origin of the proper hepatic artery [53]. These techniques have reduced the complication rate of catheter dislocation and arterial thrombosis by limiting the mobility of the catheter and the mechanical stimulation of the endothelium caused by the catheter itself. 

**Figure 1:**
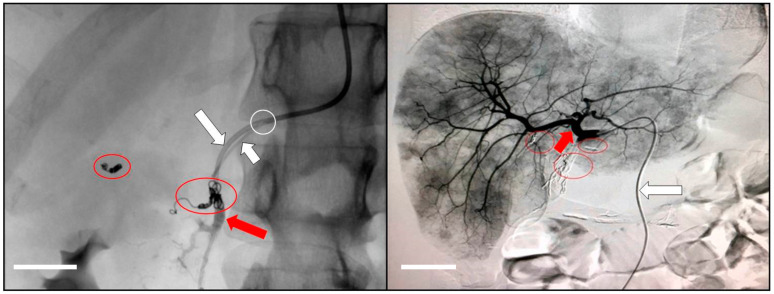
An example of the modification of the liver vascular network to avoid the reflux of cytotoxic drugs into the stomach and duodenum. Red circles indicate embolization by coils and spirals of non-target arterial branches, such as artery cystic, gastric arteries, accessory hepatic arteries, and pancreatic-duodenal arteries. The distal tip of the infusion catheter (big white arrow) is fixed within the gastro-duodenal (big red arrow) by means of vessel embolization. Therefore, the infusion hole of the catheter is facing the proper hepatic artery. The embolic agents are gradually released through a coaxial microcatheter (small white arrow) introduced into the lumen of the catheter up to the infusion hole (white circle). The small red arrow indicates proper hepatic artery at the origin of its branches. Scale bar equals 1 cm.

Thanks to the embolization by coils and spirals of non-target arterial branches (artery cystic, gastric arteries, accessory hepatic arteries, and pancreatic-duodenal arteries) healthy tissues are preserved from HAI chemotherapy, taking into account that only high spectrum cytotoxic agents are used, with no selective tropism for unhealthy sites. The embolic agents are gradually released through a coaxial microcatheter, usually of 2.7 French (Fr), which is introduced into the lumen of the catheter up to the infusion hole Figure 1.

Externally, the subcutaneous pocket is packaged, and the infusion chamber is fixed to the muscle fascia with surgical points in order to permit the administration of the drugs through an electric peristaltic pump. Finally, the proximal tip of the catheter is cut off adjacent to the infusion chamber.

**Figure 2:**
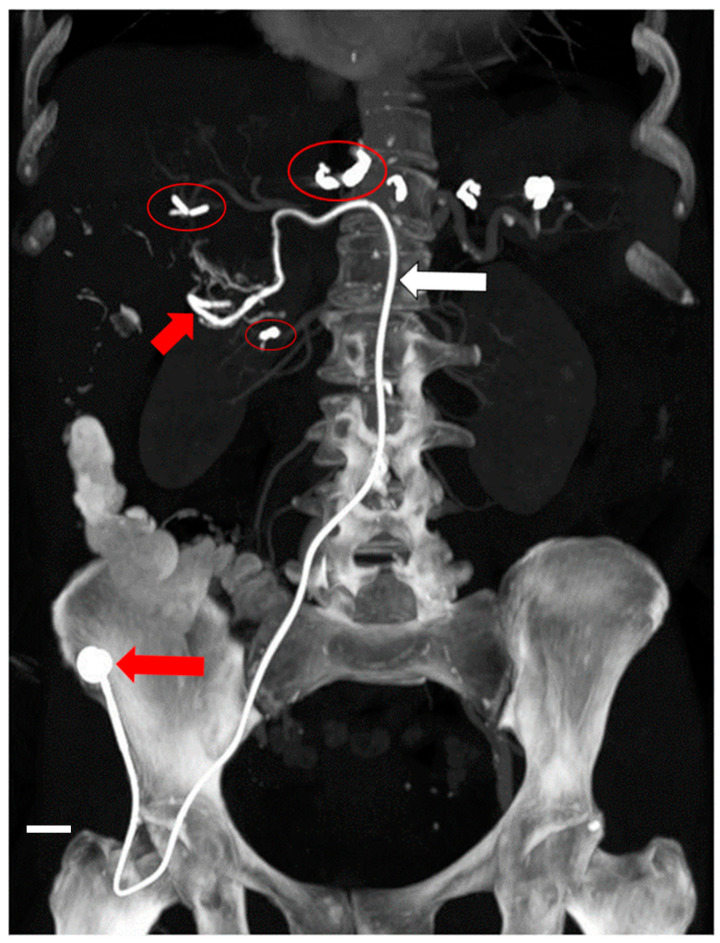
Final presentation of a hepatic arterial port-a-cath system implantation. Red circles indicate embolization by coils and spirals of non-target arterial branches such as artery cystic, gastric arteries, accessory hepatic arteries, and pancreatic-duodenal arteries. The small red arrow indicates the infusion catheter within the proper hepatic artery. The big red arrow corresponds to the infusion chamber of the port-a-cath system. Scale bar equals 1 cm.

In our Interventional Oncology, the implantation of the port-a-cath system (Celsite 5 Fr, B. Braun Medical, Saint-Cloud, France) is performed using a percutaneous femoral or subclavian approach. The catheter is slowly inserted into the gastroduodenal artery by means of a 0.016-inch hydrophilic guide (Terumo, Tokyo, Japan). The catheter of the arterial port-a-cath system, compared to the common venous port catheter, has got a 5 Fr calibre in the proximal tract, while distally it becomes more tapered with a 2.7 Fr calibre over the lateral infusion port. The distal tip of the catheter, in most cases, is fixed by means of coils and spirals, to the gastroduodenal artery or its collateral branches, such as the right gastro-epiploic artery, but always with the lateral chemotherapy infusion hole positioned within the proper hepatic artery. The proximal end of the catheter is connected, after subcutaneous tunnelling, to the infusion chamber of the port positioned and fixed to the muscle bands in the right or left iliac fossa or sub clavicular area by a surgical suture Figure 2. A final angiographic control demonstrating the correct placement of the catheter, without tension or torsion which can provoke dislocations, is always performed to verify the success of the procedure. Before each course of chemotherapy, the regular opacification of the hepatic arterial network is checked by means of the injection of a contrast medium, after percutaneous puncture of the infusion chamber with a 19 G Huber needle.

The placement procedure of hepatic arterial catheter-port system is associated with several complications, in particular subsequent to catheter dislocation, occurring in 2–44% of patients. Dislocation induces drug loss and spreading in the vicinal sites, often causing reactive gastric or duodenal mucosal lesions and irritation, responsible for abdominal pain [38,50,51]. Axillary or brachial artery accesses more frequently give rise to dislocations [54,55]. Expertise and experience in the insertion technique and catheter management have brought to its fixation by means of braided polyurethane angiographic catheters, which have significantly reduced dislocation events [56].

Bacterial infections are other complications associated with the permanently implanted catheter device and occur up to 25% of cases [57]. Inappropriate hygienic measures must be avoided by employing aseptic techniques when inserting or accessing the implantable port system, together with early usage of appropriate antibiotics [58].

Another undesired event occurring more frequently after the placement of small diameter devices, is the thrombotic occlusion of the catheter and/or the hepatic artery (4–17%) [59]. Brachial, axillary, and subclavian arteries as primary accesses should be avoided for increased rates of stroke [40,60]. Arterial thrombosis associated to percutaneous catheter placement depends on different aspects of the procedure (material and thickness of the indwelling catheters, size of the catheter compared to the target vessel lumen), as well as on toxic effects of the cytotoxic agents [50,54,59,61]. Lytic therapy with tissue plasminogen activators is considered the best strategy for acute arterial or catheter thrombosis [50,54], nonetheless prophylactic use of anticoagulation drugs is not generally considered due to its poor advantage in reducing this complication.

Bruising and formation of a small hematoma at the puncture and port pocket site is a frequent but minor problem [49]. Table 1 summarizes the different procedure-related complications and the range of frequency rates according to literature papers.

## 3. Pharmacology and Pharmacokinetics of HAI Chemotherapeutic Agents

Floxuridine (FUDR) is an antimetabolite, a pyrimidine analogue, acting as an inhibitor of cell cycle during its S-phase. It masquerades as pyrimidine-like molecules preventing the incorporation of normal pyrimidines into the DNA. The small molecule 5-fluorouracil (5-FU) is the end-product of FUDR catabolism and blocks the conversion of cytosine nucleosides into the deoxy derivatives, in addition to blocking the incorporation of the thymidine nucleotide into the DNA strand.

For years, FUDR has been the most used chemotherapeutic in HAI administration, thanks to its short half-life, its hepatic extraction rate approximatively of 95%, and its greater cancer exposure of almost 400 times than the systemic infusion [62,63]. These pharmacokinetic properties reduce systemic toxicity, particularly when combined with intravenous antitumor agents. Its medium dose in HAI infusion is 0.12 mg/kg/die via continuous infusion for 2 weeks. Unfortunately, its limited clinical indications have not consented a diffuse use in clinics, in favor of the more versatile 5-FU.

HAI 5-FU is a routine procedure for the treatment of liver metastases in interventional oncology, especially deriving from breast cancer [2]. Clinical data for HAI 5-FU are similar to FUDR at a medium dose of 1000 mg/m^2^ by continuous infusion for 44 h.

Dihydropirimidine dehydrogenase (DPD) is responsible for 5-FU catabolism, which occurs principally in the liver. DPD converts 5-FU into the less toxic dihydro-5-fluorouracil (FUH2) and, subsequently, the dihydropyrimidinase enzyme, while opening the pyrimidine ring, generates 5-fluoro-ureidopropionic acid (FUPA), which is finally transformed in the inactive α-fluoro-β-alanine (FBAL), CO_2_, and urea, eliminated in the urines within 3–4 h from intravenous administration. Only 7–20% of unmodified 5-FU is present in the plasma after intravenous administration and 90% of this quantity is eliminated in the urines within the first hour of exposure. In 6 h, no drug is present anymore, also since no plasmatic protein linking occurs. This peculiar pharmacokinetics makes the 5-FU half-life very short, within a range of 8–20 min, so that the principal administration route for this drug is via continuous infusion, both intravenous and intra-arterial. In intravenous route, the administration of an initial bolus consents to enter rapidly the therapeutic window, which is then maintained by the following continuous flow infusion.

The minor liver extraction rate for 5-FU (75–80%) with respect to its prodrug FUDR (95%), depends on its rapid metabolism by DPD, principal responsible for its low protein binding and distribution volume. 

Being 5-FU principally metabolized in the liver, continuous infusion in hepatic artery must be finely chrono-modulated in order to saturate DPD and let the active drug exert its cytotoxic activity in the DNA double-strand. Theoretically, an initial bolus of 5-FU could be able to saturate DPD, while a subsequent prompt continuous infusion could grant a major persistence in situ of the drug. 

Several studies have demonstrated that molecular defects of DPD gene (DPYD) induce deficiency of the DPD activity and are responsible for the 5-FU pharmacogenetic syndrome. The clinical manifestations of 5-FU toxicity may include fever, mucositis, stomatitis, nausea, vomiting and diarrhea, rare neurologic abnormalities, such as cerebellar ataxia and changes in cognitive function, leukopenia, neutropenia, and possible thrombocytopenia and anemia. In some cases, rare incidences of severe skin rashes may occur. Studies of full DPYD sequencing have identified a series of deleterious mutations in DPD and have contributed to the preventive selection of the patients with a greater risk for adverse effects by 5-FU, allowing an individualized approach to chemotherapy management [64,65]. The most frequent exon mutations in DPYD are IVS14 +1 G > A, D949V, c.2846A > T, and c.1679T > G, but also non-coding intronic polymorphisms (IVS5 + 18G > A, IVS6 + 139G > A, and IVS9 − 51T > G) and a synonymous mutation (c.1236G > A) have been correlated to a higher toxicity, especially in association with classical exon mutations [66].

More recently, Del Re et al. (2019) [67] have looked into this topic with a larger recruitment of 1254 patients eligible to the 5-FU treatment: They have identified c.496A > G, c.1236G > A/HapB3, c.1601G > A (DPYD*4), c.1627A > G (DPYD*5), c.1679T > G (DPYD*13), c.1896T > C, c.1905 + 1G > A (DPYD*2A), c.2194G > A (DPYD*6), and c.2846A > T mutations inducing ADRs that required dose modifications, treatment delay or discontinuation. Moreover, in the case of patients eligible to irinotecan, the UGT1A1 analysis was performed, so that the patients with UGT1A1*1 or *1/*28 genotypes were included, while patients carrying the UGT1A1*28/*28 were excluded due to the high risk of developing gastrointestinal/hematological toxicities. 

As a summary, this study englobing and generalizing also previous data has focused for the first time on a significant association between c.2194G > A (DPYD*6) and all manifestations of 5FU syndrome, in particular time to neutropenia, leukopenia, neutropenia and diarrhea, bone marrow suppression, and gastrointestinal ADRs.

Fluoropyrimidines are among the very few approved drugs to be administered both via intravenous and via intra-arterial route, together with anthracyclines and Mitomycin C. The reason for this limited arsenal of drugs to be used intra-arterially lies in the extraction rate data by the perfused organ. The highest extraction rates grant the highest local action of the drug due to its higher local concentration.

Doxorubicin and its epimer epirubicin are anthracyclines, intercalating antibiotics linking DNA and inhibiting the synthesis of nucleic acids and mitosis. In particular, they rapidly enter cells and predominantly localize on perinucleolar chromatin. After intravenous infusion, they undergo a rapid plasmatic level reduction, though accompanied by a slow urinary and biliary excretion, probably due to their high distribution volume in tissues and a low plasmatic protein linking. Biliary excretion is the principal elimination route counting for 40–50% of the administered dose within about 7 days. This huge biliary excretion related to the drug pharmacokinetics plays a key role in the local maintenance of an effective concentration in situ through the intra-arterial route. The reported hepatic extraction rate for epirubicin is 0.6 (60%) [68]. This important value, together with its slow metabolism due to its huge polycyclic structure and the absence of a specific enzyme for its degradation, has given rise over time to different attempts for augmenting its uptake through new technologies, such as adsorption on loadable microspheres of biocompatible hydrogels or conjugation with starch polymers.

A study on epirubicin infused through arterial, portal, and systemic routes has been reported in the literature: The highest concentration within the tumor site was obtained after bolus-arterial infusion and by continuous infusions, so that the artery results are better than the other routes. Differently, the highest global liver concentration resulted after portal infusion both after bolus and in 5 min [69].

The pharmacokinetic profile of the racemic doxorubicin has been studied in dogs following intra-arterial and intraportal infusion under hepatic venous isolation and charcoal hemoperfusion (HVI-CHP): The data were even more favourable with a hepatic extraction ratio of 81.2% after intra-arterial administration of 47.2% after intraportal infusion [70].

Platinum salts are N-guanine alkylating antitumor drugs with the largest spectrum of cytotoxicity and find clinical scope in a large number of neoplastic diseases, liver, and bile tract included. The commercially available Cisplatin, Carboplatin, and Oxaliplatin have been the bases of chemotherapy treatments for decades and still remain the only therapeutic strategy for many cancer diseases. In Italy, no one has been approved for intra-arterial administration, despite the fact that several world-wide studies have elaborated clinical guidelines to consider and exploit this potentially important application. 

With regards to HAI Oxaliplatin infusion, Kern in 2001 [71] published the first phase I pharmacokinetic study with increasing doses of HAI Oxaliplatin to identify the maximum tolerated dose and getting information on its pharmacokinetic profile [71]. The data underscored that Oxaliplatin, administered by HAI Oxaliplatin presented a highly differed pharmacokinetic profile, compared to the intravenous route both in terms of terminal half-life (17.8 ± 9.3 h vs. 27.3 ± 10.6 h, respectively) and AUC (17.76 ± 7.8 mcg per h/mL vs. 20.17 ± 6.97 mcg per h/mL), but was similar concerning renal clearance (135 ± 55 mL/min vs. 121 ± 56 mL/min), and elimination (49 ± 14% vs. 54 ± 20%). This finding indicated a reduced systemic diffusion of the HAI drug, associated to lower toxicity and increased availability to the target area.

Guthoff in 2003 succeeded in calculating the Oxaliplatin hepatic extraction rate after HAI administration [72,73], involving patients affected by isolated unresectable liver metastases derived from colorectal cancer. Oxaliplatin pharmacokinetics was followed through peripheral venous blood that was got before, during, and after arterial administration [72]. Comparing the AUC values after intravenous administration (161 ± 23 mcg per min/mL) with the HAI data (85.3 ± 13.7 mcg per min/mL) for the same infused dose (85 mg/m^2^), the calculated liver extraction ratio was 0.47, meaning that approximately half of the HAI administered Oxaliplatin reaches the general circulation, with a very favourable safety profile [72,73].

With respect to Oxaliplatin, Cisplatin is a less polar molecule, has a lower solubility in aqueous solution, so that its commercially available formulation has a final concentration of 1 mg/ml vs. 5 mg/ml of Oxaliplatin. For this reason, some efforts in improving the pharmaceutical aspects have been done to increase the strength of the mother solution to be used for intra-arterial administration. One of these attempts was the IA-call, a fine-powder formulation of Cisplatin, but no hepatic extraction increase was reported. 

The reported data in the literature indicate an hepatic extraction ratio for Cisplatin of 24% ± 9% after hepatic infusion [74]. 

After intravenous administration, plasmatic levels of Cisplatin decrease following a biphasic pattern, with an initial half-life of 20–50 min and a final half-life of 58–72 h. This kinetics, together with its high binding to plasmatic proteins of more than 90% does not permit a massive uptake of the drug in the liver even after intra-arterial hepatic administration, though some studies report data of effectiveness in combination therapy with intra-arterial Doxorubicin [75]. 

As for Carboplatin, no data of hepatic extraction rate have been found in the literature. Its effectiveness in clinics within the few clinical trials found, is always associated to the adsorption on loadable microsphere and not to free intra-arterial infusion.

Gemcitabine is a pyrimidine analogue, which is converted in the active forms difluorodeoxycitidin-diphosphate and triphosphate by the deoxycytidine kinase (dCK) enzyme in the cells. Gemcitabine inhibits the ribonucleotide reductase enzyme and the correct DNA synthesis. Some studies have evidenced that increased dCK levels are present in human cells of various malignancies and the selective delivery by HAI could give great advantages in sparing healthy tissues [76].

On the contrary, the deoxycytidine deaminase (dCDA) deactivates gemcitabine in inactive metabolites. Its pharmacokinetic properties after intravenous infusion are: Half-life of 42–92 min, a high distribution volume, and a low binding to plasmatic proteins. A pharmacokinetic study testing gemcitabine behavior after 24 h hepatic arterial infusion in patients with liver malignancies revealed very important and promising data in terms of liver extraction rates [77].

Surprisingly, the mean hepatic extraction ratios of gemcitabine at the 75, 135, and 180 mg/m^2^ dose level were 0.89, 0.75, and 0.55, respectively, counting for a linear decrease with an increasing dose. This trend is supposed to be caused by the saturation of dCDA in the liver or by saturation of the gemcitabine transport across the cell membrane of liver cells. To overcome this problem, a continuous infusion over 24 h can be an effective strategy for having a slow increase of drug concentrations, enzyme saturation, and drug action, exposing more cells in the S phase of cellular cycle.

Irinotecan is a camptothecin which stabilizes DNA topoisomerase I on processing the replication fork, causing single- and double-strand DNA breaks, thus inhibiting transcription and DNA replication which finally evolve in cell death. Studies on irinotecan intra-arterial infusion are present in the literature, but often associated with its adsorption on loadable microspheres. No hepatic extraction ratio has been found in the literature after free liver infusion. Despite this, it has been demonstrated that the conversion of irinotecan to its active metabolite SN38 (metabolic ratio) is increased with arterial continuous infusion due to the high content of carboxylesterase and other activating enzymes in the liver.

The panorama of all drugs tested or used in clinics for intra-arterial route administration underscores once more that the strategy of the continuous infusion is winning since it consents to exploiting different properties of the drug, such as its metabolic course and its interaction with submolecular structures, such as transporters’ saturation. In Table 2, a summary of the HAI anticancer drugs is reported.

## 4. Clinical Trials with HAI

For decades, several clinical trials have investigated the role of HAI chemotherapy for advanced hepatobiliary cancer patients in first- or subsequent-line treatments, in combination or without systemic chemotherapy. In this review, we aim at evaluating the safety in terms of AEs and the efficacy concerning disease control rate (DCR), ORR, PFS, and OS of this treatment strategy for advanced hepatobiliary cancer patients. We analyzed all prospective clinical trials of phase I, II and III, retrospective and cohort studies, as well as meta-analyses performed from 2000 to nowadays. With regards to advanced HCC, we selected only comparative trials, except for the second-line treatment, due to the elevated number of studies in the literature. Table 3 and Table 4 summarize all the clinical trials described in the following sections.

### 4.1. HAI for Unresectable Bile Ducts Carcinoma

#### 4.1.1. HAI as a First-Line Treatment without Systemic Therapy

In 2002, Tanaka et al. [78] reported the clinical results for 11 never treated patients that underwent HAI with 5-FU, every 1–2 weeks using three different regimens, by means of a catheter and a port system percutaneously implanted. In addition, 36.3% of the enrolled patients had PVTT. Authors reported a DCR of 82% and a mean survival of 26 months. With regards to toxicity, 27% of patients experienced grade 3–4 AEs such as cholangitis and pancytopenia. 

In 2009, Jarnagin et al. [79] in a phase II study analyzed HAI FUDR at a dose of 0.16 mg/kg × 20/pump flow rate, administered through a surgically implanted infusion pump in 34 patients (26 with ICC and 8 with HCC). This clinical trial demonstrated a DRC of 88.2% higher in ICC patients rather than HCC ones and a median OS of 29.5 months. Grade 3–4 AEs, such as elevated bilirubin levels, abdominal pain, and diarrhea were reported in 20% of patients, while 6% experienced technique-related complications, such as infection and pump dislocation.

In 2011, Inaba et al. [80] investigated in a phase I/II study the efficacy and safety of HAI Gemcitabine at three dosage levels of 600, 800, and 1000 mg/m^2^ in untreated patients. The administration was performed via the port system in 30 min on day 1, 8, and 15 every 4 weeks for 5 cycles. Authors enrolled 16 and 13 patients during phase I and II, respectively. DCR was 69% with a median OS of 12.1 months. Among grade 3 AEs, 20% mielo-suppression, 4% elevated gamma-glutamyl transpeptidase (GGT) and/or elevated aspartate aminotransferase (AST), 4% elevated alanine aminotransferase (ALT), and 1% elevated bilirubin were observed. The only grade 4 event was elevated bilirubin in 1% of patients. Technical difficulties in the placement of the port-a-cath system in 17% (catheter obstruction in 10% of patients, port damage in 7% of patients) and hepatic artery occlusion in 3% of patients were observed, respectively.

In 2013, Sinn et al. [81] investigated biweekly HAI Oxaliplatin at the dosage of 85 mg/m^2^ in 120 min infusion and 5-FU (600 mg/m^2^ mixed with natrium folinate 170 mg/m^2^ in 120 min infusion) in 37 patients. DCR was 64.9% and the median OS was 13.5 months. Thrombosis (13.5%), dislocation (10.8%), and infection (8.1%) were pump-related events. Grade 3–4 AEs including haematological cytopenia (16%), increasing enzymes liver levels (18.9%), and severe abdominal pain (8%) were reported.

In 2013, Kasai et al. [82] analyzed HAI 5-FU (250 mg/day for 5 h on day 1–5 of every week, for 4 weeks) by means of an intra-arterial catheter and port system implanted subcutaneously, combined with subcutaneous PEG-IFNalpha-2b (50–100 µg on day 1 of every week, for 4 weeks) in 20 patients (five of them with associated extrahepatic disease). Each course was repeated every 4 weeks. DCR was 90% and the median OS was 14.6 months. No complications regarding the HAI technique were described. Toxicity was mild without grade 4 events, but 15% of patients experienced grade 3 haematological AEs. 

In 2015, Massani et al. [83] enrolled 11 patients who underwent HAI 5-FU (7 mg/kg in continuous infusion for 48 h) and Oxaliplatin (100 mg/m^2^ in continuous infusion for 5 h) after the placement of a HAI pump. Each course was repeated every 2 weeks. DCR was 63.6% and the median OS was 15.3 months, while no data on toxicity were reported.

In 2016, Wang et al. conducted a phase II study [84] for the treatment of 37 patients, with HAI Oxaliplatin (40 mg/m^2^ for 2 h) plus 5-FU (800 mg/m^2^ for 22 h on day 1–3) every 3–4 weeks and for a maximum of 6 courses, followed by Capecitabine maintenance. HAI administration was executed by means of an implanted port-a-cath system. DCR was 89.2%, while the median PFS and median OS were 12.2 and 20.5 months, respectively. Severe haematological cytopenia was reported in 16.2% patients and grades 3–4 liver enzyme elevation was observed in 8.1% patients. With regards to complications of HAI, hepatic artery occlusion occurred in 2.7% of the patient population and caused treatment discontinuation, while extrahepatic gastric infusion occurred in 10.8% of patients. Finally, 5.4% of patients experienced severe abdominal pain, which was effectively managed with HAI of the lidocaine salt solution.

In 2018, Higaki et al. [85] tested HAI IA-call (a fine-powder formulation of Cisplatin) plus oral S-1 compared with other treatments such as radiation therapy, trans-arterial chemoembolization, and systemic chemotherapy in 12 patients. The IA-call plus S-1 regimen consisted of IA-call (65 mg/m^2^, administered into the hepatic artery) on day 1 and oral S-1 (60 mg/m^2^/day) on days 1–28, every 42 days in a repeated cycle. A catheter was placed within the femoral artery and introduced into the hepatic artery under angiographic guidance. DCR was 58.3%, OS was significantly longer in the patients receiving the HAI treatment than the other approaches (10.1 months vs. 4.0 months, *p* = 0.01). The major toxic effect was grade 3 anemia, occurring in 4.5% of patients.

#### 4.1.2. HAI as a First-Line Treatment Combined with Systemic Therapy

In 2004, Cantore et al. [68] in a phase II study enrolled 30 untreated patients that received Epirubicin (50 mg/m^2^) and Cisplatin (60 mg/m^2^) *in bolus* through a catheter into the hepatic artery using the Seldinger technique. They also received continuous intravenous infusion of 5-FU (200 mg/m^2^ from day 1 to 14). The complete regimen was repeated every 3 weeks. DCR was 80%, the median PFS and OS were 7.1 and 13.2 months, respectively and no procedure-related AEs were observed systemically. In addition, 37% of patients experienced severe AEs, such as acute pancreatitis, mucositis, and leukopenia. 

In 2007, Mambrini et al. [86] in a phase II study combined HAI epirubicin (50 mg/m^2^) and Cisplatin (60 mg/m^2^) in bolus with oral Capecitabine (1000 mg/m^2^ bid from day 2 to 15) every 3 weeks, in 20 patients. Among them, only 10% of patients have already received the prior treatment. The DCR was 79%, while the median PFS and OS were 11.6 and 18 months, respectively. In terms of toxicity, no angiographic procedure-related AEs were documented, 5% of patients died due to severe diarrhea while the other patients experienced minimal toxicity. 

In 2011, Kemeny et al. [87] evaluated the addition of weekly Bevacizumab at 5 mg/kg to HAI FUDR at the dose of 0.16 mg/kg × 30/pump flow rate for a 14 day infusion, every 4 weeks, through a placed pump, in 22 patients (18 with ICC and 4 with HCC). Excitedly, this trial showed a DCR of 100%, a median PFS and OS of 8.5 and 31.1 months, respectively. However, this study was prematurely closed, due to the increased biliary toxicity (24% of patients experienced uncontrolled bilirubin elevation) related to the association with Bevacizumab. 

In 2015, Kostantinidis et al. [88] retrospectively analyzed 104 patients who had underwent combined HAI and systemic chemotherapy (78/104) or systemic chemotherapy alone as a control arm (26/104). HAI was executed by means of a surgically implanted pump and consisted of a continuous infusion of FUDR for 14 days every 4 weeks. Systemic chemotherapy was administered during 2 weeks free from HAI and consisted of the Gemcitabine-, Irinotecan- or 5-FU-based regimen. Patients in the control group often received the Gemcitabine-based combination regimen. RR in the combined group was better than the control group, but no statistical significance (59% vs. 39%; *p* = 0.11) was reported. The OS for the experimental group was longer than the control group (30.8 months vs. 18.4 months; *p* < 0.001), while no data on toxicity were reported.

In 2019, Cerker et al. [89] evaluated clinical results from the combination of HAI FUDR ((0.12 mg/kg × kg × 30)/pump flow rate) with systemic Gemcitabine (800 mg/m^2^) and Oxaliplatin (85 mg/m^2^) in 38 patients. All patients underwent surgical HAI pump placement. This phase II clinical trial showed that 84% of the patients achieved DCR at 6 months. The median PFS was 11.8 months while the median OS was 25.0 months. The most common grade 3 toxic effects included alterations in the liver function test in 42% of patients, while 11% had grade 4 AEs requiring removal from the study (one portal hypertension, two gastroduodenal artery aneurysms, one infection in the pump pocket). 

#### 4.1.3. HAI as a Second-Line Treatment

In 2014, Ghiringhelli et al. [90] evaluated HAI Gemcitabine (1000 mg/m^2^ in 30 min infusion) plus Oxaliplatin (100 mg/m^2^ in 2 h infusion) every 2 weeks as a second-line treatment in 12 patients that have previously received at least one line of systemic chemotherapy (Gemcitabine or Oxaliplatin). Percutaneous implantation of the port catheter was performed for HAI. The DCR and median OS were 91% and 9.1 months, respectively. No HAI procedure-related events were observed. Only 16.7% of patients developed grade 3–4 toxicities, including haematological cytopenia and 16% had anaphylactic reaction to Oxaliplatin.

### 4.2. HAI for Unresectable HCC

#### 4.2.1. HAI as a First-Line Treatment without Systemic Therapy

In 2017, Kodama et al. [91] conducted a retrospective cohort study with 68 patients treated with subcutaneous IFN + HAI 5-FU, by means of an implanted port-a-cath system, associated with 3 D-conformal radiotherapy (the median radiation dose was 39 Gy (range, 30–45 Gy) delivered in 13 fractions (range, 10–15)) and 40 patients treated with Sorafenib. The median OS and PFS were significantly longer in the HAI chemotherapy + RT group than in the Sorafenib group (9.9 months vs. 5.3 months; *p* = 0.002; 3.9 months vs. 2.1 months, respectively; *p* = 0.048). The grade 3/4 AEs reported in the HAI chemotherapy + RT group were haematological events (12%), increased AST or ALT (2.7%), increased bilirubin level (5.5%), diarrhea, general fatigue (2.7%), and infection of the port system (2.7%). In the Sorafenib group, the following grade 3/4 AEs were observed: Increased AST or ALT (8.3%), increased bilirubin level (5.5%), diarrhea and general fatigue (13.9%), gastrointestinal bleeding (2.7%), high blood pressure (2.7%), and hand-foot syndrome (2.7%).

In 2018, Lyu et al. [92] evaluated in a phase II study the comparison between 180 patients treated with HAI FOLFOX-6 vs. 232 patients treated with Sorafenib (400 mg twice daily). HAI administration was performed using a micro-catheter placed into the hepatic artery. This trial reported that the median PFS and OS were significantly longer in the HAI group (*p* < 0.001 for each), with a PFS of 7.1 months vs. 3.3 months and OS of 14.5 months vs. 7 months in the HAI FOLFOX-6 group and Sorafenib group, respectively. In the Sorafenib group, the frequency of severe AEs was 12% in contrast with 9% in the HAI group. The most frequent grade 3–4 AEs were leukopenia, fatigue, elevated AST and/or ALT, abdominal pain, hypoalbuminemia in the HAI group, while the hand-foot skin reaction, diarrhea, and haematological alterations in the Sorafenib group.

In 2018, Kawaoka et al. [93], in a retrospective, comparative cohort study, tested HAI chemotherapy vs. Sorafenib in 177 patients. HAI chemotherapy (*n* = 136) consisted of 1. Cisplatin (6 mg/kg per day at days 1–5 and 8–12) plus 5-FU (300 mg/m^2^ per day in continuous infusion over 24 h during days 1–5 and 8–12 in each course) or 2. 5-FU in combination with subcutaneous interferon injection, recombinant IFNα-2b (total dose: 36 MU) or natural IFN-α (5 MU for a total dose of 60 MU) at days 1, 3, and 5 of each week. HAI administration was performed employing a micro-catheter placed into hepatic artery. Each chemotherapy course was repeated after 2 or 4 weeks. Sorafenib (*n* = 41) was administered at a 800 mg/die dosage. DCR were 92.3% vs. 89.9%, RR were 30.9% vs. 4.8%, OS were 14 months vs. 7 months (*p* = 0.005) in the HAI group and Sorafenib group, respectively. Among HAI patients, 2.2% experienced grade 3–4 liver failure, while 4.9% of patients developed grade 3–4 worsening of performance status in the Sorafenib group. 

In 2018, Choi et al. [94] in a randomized, prospective, comparative trial evaluated HAI Cisplatin (60 mg/m^2^ for 2 h on day 2) plus 5-FU (500 mg/m^2^ for 5 h on days 1–3), by means of an implanted port-a-cath system, every 3–4 weeks (*n* = 58) vs. Sorafenib (800 mg/die) (*n* = 29). The ORR was 27.6% vs. 3.4% (*p* = 0.001), OS was 14.9 months vs. 7.2 months (*p* = 0.012); and TTP was 4.4 months vs. 2.7 months (*p* = 0.010) in the HAI chemotherapy and Sorafenib groups, respectively. Severe AEs were hyperbilirubinemia (44.8%), AST elevation (34.5%), ascites (13.8%), and catheter-related complications (3.4%) in the HAIC group and hyperbilirubinemia (34.5%), hand-foot syndrome (31.0%), and AST elevation (27.6%) in the Sorafenib group.

A 2019 recent meta-analysis [95] evaluated the efficacy and safety of HAI chemotherapy (*n* = 1006) vs. Sorafenib (*n* = 773) in patients affected by advanced HCC and collected from 14 recent clinical trials. Clinical data reported that HAI chemotherapy was associated with significantly longer DCR, ORR, PFS, and OS rather than Sorafenib. Furthermore, Sorafenib was associated with a higher frequency of AEs, such as hypertension, fatigue, dermatological, and gastrointestinal disorders.

In 2020, Ueshima et al. [96], in a retrospective, comparative, cohort study, compared HAI chemotherapy (*n* = 541) vs. Sorafenib (*n* = 1465). This study evidenced that HAI chemotherapy significantly improves OS in patients with PVTT but without extrahepatic disease with respect to Sorafenib (10.1 months vs. 9.1 months, respectively). The clinical trial did not show a significant difference in OS between patients without both PVTT and extrahepatic disease (12.2 months vs. 15.4 months for the HAI and Sorafenib groups, respectively). The most common HAI technique-related AEs were catheter occlusion (2.3%) and abdominal pain (2.1%). On the other hand, the most frequent Sorafenib-related events were general disorders (3.6%), rash (2.2%), hand-foot-skin reaction (2.2%), and anorexia (2.1%).

In 2020, Ahn et al. [97], in a retrospective and comparative trial, investigated HAI Cisplatin (60 mg/m^2^ for 1 day) and 5-FU (500 mg/m^2^ for 3 days) every 4 weeks (*n* = 38 patients) using the Seldinger method implantation vs. Sorafenib (400 mg twice daily) (*n* = 35 patients). Baseline characteristics were similar between the two groups, except the presence of solid organ metastasis (46% vs. 5.3%, *p* < 0.001). The authors reported that the median OS was not significantly different between the groups (6.4 months vs. 10.0 months, *p* = 0.139), while TTP was significantly longer in the experimental group (6.2 months vs. 2.1 months, *p* = 0.006) as well as the DCR (76% vs. 37%, *p* = 0.001). Moreover, when patients with extrahepatic solid organ metastasis were excluded, the median OS time was 8.8 months vs. 11.1 months (*p* = 0.097), TTP was 1.9 months vs. 6.0 months (*p* < 0.001), and DCR was 53% vs. 81% (*p* = 0.030). More hematologic AEs occurred in the HAI group, while more constitutional complications were observed in the Sorafenib group. In addition, 13.2% of patients discontinued the treatment due to catheter-related complications (catheter occlusion and/or infection). 

A 2020 recent systematic review [98] evaluated the efficacy and safety of HAI chemotherapy vs. Sorafenib. In addition, 417 patients were included from 43 studies, showing that HAI chemotherapy was associated with significantly longer DCR, PFS, and OS than Sorafenib. HAI chemotherapy was associated to more grade 3–4 haematological events, while grade 3–4 aspartate aminotransferase rising, diarrhea, and hand-foot syndrome were more frequent in the Sorafenib group.

#### 4.2.2. HAI as a First-Line Treatment Combined with Systemic Therapy

In 2018, Kudo et al. [99] conducted a phase III trial (SILIUS), testing the combination of HAI (Cisplatin 20 mg/m^2^ on day 1 through an implanted catheter system) with Sorafenib compared to Sorafenib alone. In addition, 206 patients were enrolled (103 were assigned to the combination group and the others to the Sorafenib one). The addition of HAI to Sorafenib did not significantly increase OS (11.8 months vs. 11.5 months; *p* = 0.955) as well as the median PFS that was similar in both groups (4.8 months vs. 3.5 months; *p* = 0.051). However, the combination significantly improved median TPP (5.3 months vs. 3.5 months; *p* = 0.004) and ORR (37% vs. 18%; *p* = 0.003). Severe AEs were more frequent in the combination group, including haematological events and anorexia.

In 2019, Kondo et al. conducted a multi-centre randomized phase II study [100] called SCOOP-2 trial on the comparison between HAI chemotherapy, using the Seldinger method implantation, plus the Sorafenib group (*n* = 35 patients) and Sorafenib alone group (*n* = 33 patients). Specifically, patients in the experimental group underwent HAI Cisplatin followed by Sorafenib at the starting dosage of 400 mg twice daily in both groups. This trial reported that the sequential treatment did not improve the survival benefit than Sorafenib alone. No unexpected AEs related to HAI or Sorafenib were reported in both groups. However, this study was probably underpowered, due to the low number of enrolled patients compared to ones required by the study design and that the HAI efficacy could have been halted due to the clinical progressive disease events based on the alpha-fetoprotein and des-gamma carboxyprothrombin levels. 

In 2019, He et al. [101,102] in a randomized, open-label clinical trial, evaluated the efficacy and safety of HAI FOLFOX, by means of an intra-arterial catheter, plus sorafenib compared to Sorafenib alone for 247 patients. The combination group received HAI Oxaliplatin (85 mg/m^2^), leucovorin (400 mg/m^2^), 5-FU bolus (400 mg/m^2^) on day 1, and 5-FU infusion (2400 mg/m^2^) for 46 h, every 3 weeks. Sorafenib was administered at a 400 mg dosage twice daily in each group. For 247 patients, the median OS was significantly longer in the combination group (13.37 months vs. 7.13 months; *p* < 0.001) as well as RR (40.8% vs. 2.46%; *p* < 0.001), and median PFS (7.03 months vs. 2.6 months; *p* < 0.001). Severe AEs were more frequent in the combination group, such as haematological events (22% vs. 7.5%) and vomiting (6.5% vs. 1%).

A 2020 recent meta-analysis [103] analyzed the efficacy and safety of HAI chemotherapy plus Sorafenib compared to Sorafenib as a single agent. In addition, 726 patients were included from five studies reporting that HAI (one with Oxaliplatin, 5-FU, and Leucovorin; two with Cisplatin; two with Cisplatin and 5-FU) plus the Sorafenib group was associated with a significantly longer OS and higher overall RR than other therapies. In particular, multiple chemotherapies significantly improved ORR than single-agent chemotherapy. Moreover, the combination group showed a significant higher risk of haematological AEs. 

#### 4.2.3. HAI as a Second-Line Treatment

In 2014, Terashima et al. [104] evaluated the safety and efficacy of HAI chemotherapy as a second-line treatment after Sorafenib, enrolling 27 patients. HAI chemotherapy, through an implanted catheter system, consisted of Cisplatin (20 mg/m^2^ per day for 10 min) plus the continuous infusion over 24 h of 5-FU (330 mg/m^2^ per day) from days 1–5 and 8–12 and the subcutaneous administration of pegylated interferon α-2b (1 μg/kg) on days 1, 8, 15, and 22. Each course lasted 28 days, followed by 2 weeks of rest. DCR was 62.9%, the median PFS and OS were 4 and 7.6 months, respectively. Grade 3–4 AEs were reported, such as neutropenia and thrombocytopenia (51.9% and 48.1%, respectively) and device-related complications were observed in 18.5% of patients.

## 5. Discussion

ICC and HCC are the most frequent primary liver cancer and their frequency and mortality have been increasing in the last decades. These tumors often present at an advanced stage, not amenable to surgery, with limited treatment options. However, even the respectable disease that underwent surgery frequently recurs with limited survival [12,105].

Nowadays, the gold standard treatment for advanced biliary cancers corresponds to the combination of gemcitabine and platinum-based chemotherapy, which offers a modest benefit in terms of OS compared to gemcitabine monotherapy (11.7 months vs. 8.1 months, respectively) [18]. The administration of biological agents such as cetuximab did not improve the survival of these patients [106]. Furthermore, new mutations have been identified with the aim to find new targeted therapies [107]. 

With regards to the advanced HCC treatment, Lenvatinib is a new first-line therapy that demonstrates to be non-inferior to Sorafenib in terms of efficacy [13]. Moreover, Cabozantinib and Regorafenib are new available second-line therapies improving survival benefits after the Sorafenib failure [14,15]. However, the enrolled patients in the relative clinical trials have a good liver function and well-reserved performance status as well as in the Sorafenib trials. Therefore, these drugs have limited applicability in real practice. 

The poor prognosis and the few available therapies enriched of severe AEs lead to the necessity of exploring new more effective treatment strategies for these types of tumors.

In the case of primary and secondary liver tumors, the hepatic artery becomes the main source of blood favoring tumor growth [4]. This is the reason why HAI delivering chemotherapeutic agents selectively within liver parenchyma, through a catheter or pump, is able to attack directly local disease, while sparing healthy liver and minimizing systemic toxicity [108]. Moreover, the administration of anticancer drugs with the high hepatic extraction rate could also favor the direct killing action on cancer cells and minimize systemic AEs.

Most of the studies collected in this review have several limitations: non-randomized retrospective design, a relatively small number of patients, the administration of different HAI chemotherapeutic agents, as well as the combination of HAI with a great heterogeneity of systemic agents. However, despite these limitations, the presented data show favorable results in terms of safety and efficacy for HAI chemotherapy, with respect or in alternative to the gold standard treatment, in advanced ICC and HCC, even when they are combined with systemic treatments. Therefore, HAI chemotherapy may be an alternative or an integrative treatment option for advanced hepatobiliary cancers. 

Hence, further and larger prospective, randomized, multi-center studies, with well-defined inclusion criteria and treatment strategies, are required to confirm the above presented data. In addition, it is necessary to define what patients could better benefit of this type of treatment and what drugs should be preferred, in consideration of the different pharmacokinetic and pharmacodynamic profiles. In our opinion, it is necessary that future studies are carried out in several specialized centers where these techniques are performed by professionals with expertise in the specific field of loco-regional treatments. In detail, skilled interventional oncologists, medical oncologists with expertise in systemic and loco-regional chemotherapies, and pharmacists with specialized knowledge in HAI chemotherapeutic agents. With regards to patient populations, we retain that advanced ICC and HCC patients, with or without PVTT and in the absence of extrahepatic disease, should be enrolled in two different double-blind, prospective, randomized, multi-center clinical trials. Based on the presented literature data, we propose that the experimental group of ICC patients receive HAI Oxaliplatin and systemic Gemcitabine compared to the control group treated with systemic Gemcitabine plus platin-based chemotherapy. On the other hand, the experimental group of HCC patients should receive HAI 5-FU with or without Sorafenib compared to the control group treated with Sorafenib alone.

## 6. Conclusions

HAI chemotherapy is a locoregional technique that has been evaluated for the treatment of advanced primary liver tumors in several clinical trials. Some of them explored this type of therapy in combination with systemic treatments. Most of the clinical trials reported in this review shed light on the important improvement of disease control and survival in these patients. Moreover, drugs used for HAI have proven to be safe even when they were combined with systemic treatments. 

Pharmaceutical and pharmacokinetic properties are extremely important in order to select the best drugs to be used locoregionally, in particular in relation to their values of hepatic extraction rate. In the same way, the kinetic granted by continuous infusions should be taken into account more than the administration in bolus, since it can lead to a better steady-state of the local drug concentration, with a critical reduction of systemic toxicity.

The promising outcomes, although preliminary, in different clinical trials support the safety and efficacy of HAI chemotherapy for advanced primary liver tumors. For this reason, a randomized, phase III clinical trial including several centers with expertise in these approaches, with an adequate selection of patients to undergo this treatment, are needed to confirm the data already published.

## Figures and Tables

**Table 1:** Procedure-related complications and their frequencies.

Complication	Range of Frequency Rates
Catheter dislocation	2–44%
Infection	4–25%
Thrombotic occlusion of catheter or hepatic artery	4–17%
Bruising and hematoma at the puncture and port pocket site	5–16%

**Table 2:** Anticancer drug clinically used and/or tested for HAI administration.

Anticancer Drug	Pharmacologic Class	Hepatic Extraction Rate	References
Floxuridine	Antimetabolite (pyrimidine analogue)	95%	Kemeny et al. (1986) [63]
5-Fluorouracile	Antimetabolite (pyrimidine analogue)	75–80%	Technical Sheet [64,65]
Doxorubicin	Intercalating antibiotic (anthracycline)	Up to 81.2% (in dogs)	Iwasaki et al. (1998) [70]
Epirubicin	Intercalating antibiotic (anthracycline)	60%	Cantore et al. (2005) [68]
Oxaliplatin	Alkylating agent (platinum salt)	47%	Guthoff et al. (2003) [72]
Cisplatin	Alkylating agent (platinum salt)	24 ± 9%	Campbell et al. (1983) [74]
Gemcitabine	Antimetabolite (nucleoside analogue)	55–89% (dose-dependent)	Von Riel et al. (2009) [77]
Irinotecan	Topoisomerase I inhibitor (camptothecin)	N.D.	-

**Table 3:** Compilation of references included in the review of literature that evaluated HAI chemotherapy in patients with advanced cholangiocarcinoma.

References	Type of Study	No. of Pts	HAI Chemotherapy	Systemic Chemotherapy-Associated	DCR (%)	mPFS (mo.)	mOS (mo.)
Tanaka et al. (2002) [78]	I	11	5-FU	No	82	n.e.	26 ^a^
Jarnagin et al. (2009) [79]	II	34	FUDR	No	88.2	n.e.	29.5
Inaba et al. (2011) [80]	I/II	16/13	Gemcitabine	No	69	n.e.	12.1
Sinn et al. (2013) [81]	II	37	Oxaliplatin and5-FU	No	64.9	n.e.	13.5
Kasai et al. (2014) [82]	II	20	5-FU	No	90	n.e.	14.6
Massani et al. (2015) [83]	Review/II	11	5-FU and Oxaliplatin	No	63.3	n.e.	15.3
Wang et al. (2016) [84]	II	37	Oxaliplatin plus 5-FU	No	89.2	12.2	20.5
Higaki et al. (2018) [85]	Pilot Study	12	(IA-call and oral S-1)	No	58.3	n.e.	10.1
Cantore et al. (2004) [68]	II	30	Epirubicin and Cisplatin	5-FU	80	7.1	13.2
Mambrini et al. (2007) [86]	II	20	Epirubicin and Cisplatin	Capecitabine	79	11.6	18
Kemeny et al. (2011) [87]	II	22	FUDR	Bevacizumab	100	8.5	31.1
Kostantinidis et al. (2015) ^c^ [88]	Retrospective	104	FUDR	Gemcitabine-, irinotecan- or 5-fluorouracil-based regimen	59 vs. 39 ^b^	n.e.	30.8 vs. 18.4 *
Cerker et al. (2019) [89]	II	38	FUDR	Gemcitabine and Oxaliplatin	84	11.8	25
Ghiringelli et al. (2014) [90]	II	12	Gemcitabine and Oxaliplatin	No	91	n.e.	9.1

^a^ Mean survival; ^b^ ORR; Pts: patients; n.e.: Not evaluated; ^c^ comparative study; mo.: Months; * significant statistical difference.

**Table 4:** Compilation of references included in the review of literature that evaluated HAI chemotherapy in patients with advanced HCC.

References	Type of Study	No. of Pts	HAI Chemotherapy	Systemic Treatment Associated	DCR (%)	mPFS (mo.)	mOS (mo.)
Kodama et al. (2017) [91]	Retrospective cohort study	68	HAI chemotherapy associated with 3 D-conformal radiotherapy	No	n.e.	3.9 vs. 2.1 *	9.9 vs. 5.3 *
Lyu et al. (2018) [92]	II	412	FOLFOX-6	No	n.e.	7.1 vs. 3.3 *	14.5 vs. 7 *
Kawaoka et al. (2015) [93]	Retrospective cohort study	177	Cisplatin plus 5-FU or 5-FU in combination with subc. IFN infection or 5 MU natural IFN-α	No	92.3 vs. 89.9	n.e.	14 vs. 7
Choi et al. (2018) [94]	II	58	Cisplatin plus 5-FU every 3–4 weeks	No	27.6 vs. 3.4 ^a,^*	4.4 vs. 2.7 ^b,^*	14.9 vs. 7.2 *
Zhuang et al. (2019) ^1^ [95]	Meta-analysis	1779	Several regimens	No	*	*	*
Ueshima et al. (2020) [96]	Retrospective cohort study	2006	No data	No	n.e.	n.e.	10.1 vs. 9.1 *
Ahn et al. (2020) [97]	Retrospective cohort study	73	Cisplatin and 5-FU	No	76 vs. 37 *	6.2 vs. 2.1 ^b^	6.4 vs. 10
Liu et al. (2020) ^1^ [98]	Meta-analysis	417	Several regimens	No	*	*	*
Kudo et al. (2018) [99]	III	206	Cisplatin	Sorafenib	37 vs. 18 ^c^	4.8 vs. 3.5/5.3 vs. 3.5 ^b,^*	11.8 vs. 11.5
Kondo et al. (2019) [100]	II	68	HAI Cisplatin followed by Sorafenib	Sorafenib	-	-	-
He et al. (2019) [101,102]	II	247	FOLFOX	Sorafenib	40.8 vs. 2.46 ^c,^*	7.03 vs. 2.6 *	13.37 vs. 7.13 *
Ouyang et al. (2020) ^1^ [103]	Meta-analysis	726	Several regimens	Sorafenib	*	n.e.	*
Terashima et al. (2014) ^2^ [104]	I/II	27	Cisplatin plus 5-FU and the subc. administration of pegylated interferon α-2b	No	62.9	4	7.6

^a^ ORR; ^b^ TTP; ^c^ RR; ^1^ meta-analysis; ^2^ second-line treatment; * significant difference.
